# Identification and selection of healthy spermatozoa in heterozygous carriers of the Phe508del-variant of the CFTR-gene in assisted reproduction

**DOI:** 10.1038/s41598-022-05925-1

**Published:** 2022-02-03

**Authors:** Julie De Geyter, Sabina Gallati-Kraemer, Hong Zhang, Christian De Geyter

**Affiliations:** 1 Department of Medical Genetics, University Hospital, University of Basel, Schönbeinstrasse 40, 4031 Basel, Switzerland; 2 Reproductive Medicine and Gynecological Endocrinology (RME), University Hospital, University of Basel, Vogesenstrasse 134, 4031 Basel, Switzerland; 3 Division of Human Genetics, University Hospital of Berne, University of Berne, Freiburgstrasse 18, 3010 Bern, Switzerland; 4 Department of Biomedicine (DBM), University Hospital, University of Basel, Hebelstrasse 20, 4031 Basel, Switzerland

**Keywords:** Biological techniques, Developmental biology, Genetics, Medical research, Urology

## Abstract

The pathogenic variant Phe508del of the CFTR-gene is the most frequent cause of cystic fibrosis (CF). Whereas male CF-patients are infertile due to bilateral agenesis of the efferent ducts, the fertility status of male heterozygous carriers is uncertain. We aimed at demonstrating the involvement of the CFTR-ion channel during sperm capacitation and to potentially select variant-free spermatozoa in heterozygous carriers of the CFTR-variant using flow cytometry (FC). Using FC and sorting, single cell polymerase chain reaction, immuno-fluorescent staining an experimental study was performed on nine fertile semen donors and three heterozygous infertile men carrying the Phe508del gene variant. Chemical inhibition of CFTR interfered with sperm capacitation. Most viable spermatozoa of heterozygous carriers of the Phe508del variant of the CFTR-gene show immune-fluorescent CFTR. Sperm capacitation in Phe508del carriers was similar to that in healthy semen donors. Distribution of the Phe508del allele in trio data of CF-affected families corresponded to the expected recessive inheritance pattern. Infertility in Phe508del heterozygous men is unlikely to be caused by the pathogenic variant although some contribution cannot be excluded. Normal sperm capacitation in carriers of pathogenic variants of the Phe508del-gene may in part explain the high prevalence of a potentially lethal recessive disorder.

## Introduction

Cystic fibrosis (CF) is the most common autosomal recessive genetic disease with a mean prevalence of 0.737 per 10,000 individuals in Europe^1^. CF is caused by a few of more than 2000 different variants in the *CFTR*-gene encoding the CF transmembrane conductance regulator (CFTR) ion channel^2^. The *CFTR*-gene is located on the long arm of chromosome 7 at position q31.2. The most common variant causing CF is Phe508del (formerly known as ΔF508) encoding the abnormal protein p.Phe508del^3^. Phe508del is most common among population living in Central and Northern Europe and in the Basque countries^4–6^, whereas other pathogenic variants may occur in other ethnicities as well^7^. The pathogenic variant Phe508del represents a deletion of 3 base pairs resulting in the loss of codon 508 encoding phenylalanine and in consequence in altered phosphoregulation of the CFTR-protein resulting in the increased retention of p.Phe508del in the endocytoplasmic reticulum, in rapid degradation at the cell surface and in reduced recycling of p.Phe508del to the cell surface^8^. Abnormal CFTR, such as p.Phe508del, disturbs the intracellular transport of both chloride (Cl^−^) and bicarbonate (HCO3^−^) into epithelial cells disrupting the outward flow of water producing thickened mucus, which impairs function of lungs and digestive organs potentially becoming infected repeatedly. Affected individuals suffer from progressive lung disease, chronic sinusitis, pancreatic insufficiency, and malabsorption. Male CF patients are infertile presenting with obstructive azoospermia due to congenital bilateral absence of the efferent ducts (CBAVD)^9^. Some female CF patients present with reduced fertility due to thickened cervical mucus within the genital tract preventing the upward migration of spermatozoa and local lack of bicarbonate ions impairs fertilisation^10,11^ but a majority achieves pregnancy naturally. Novel CFTR modulating medication will likely raise the number of couples carrying pathogenic variants willing to achieve pregnancy^12,13^.

Conflicting reports have been published with respect to the fertility of heterozygous male individuals carrying one or more pathogenic variants of the *CFTR*-gene^14–16^ (for review see^17^). The CFTR-ion channel is known to be involved in the capacitation of sperm, which consists of hyperactivated motility, increased fluidity of the membrane and an intracellular accumulation of ions, including chloride and bicarbonate. Capacitation is an essential process through which spermatozoa acquire fertilizing capacity^18^. The involvement of CFTR during capacitation has been reported to be required for normal sperm function^19^.

Because spermatozoa are haploid, half of the spermatozoa of heterozygous men carrying the Phe508del mutation should have an impaired capacitation. Flow cytometry (FC) and sorting (FACS) may potentially be used to select human spermatozoa with distinct physiological characteristics^20,21^. FC allows the visualisation of the transmembrane transport of chloride and bicarbonate ions^22^. We aimed at demonstrating the involvement of the CFTR-ion channel during capacitation of human spermatozoa and, if so, at selecting pathogenic variant-free spermatozoa in infertile men heterozygous for the Phe508del, that can be used in assisted reproduction.

## Results

### Discrimination between capacitated and non-capacitating spermatozoa based on ion passage through the sperm membrane

Capacitation of sperm refers to the changes that spermatozoa must undergo in order to be able to fertilize an egg. The early stages of capacitation include a change in the movement pattern, characterized by hyperactivated velocity. Aiming at verifying the degree of capacitation of the washed semen the semen samples of eight normal semen donors were incubated in non-capacitating medium and in capacitating culture medium, respectively. The degree of capacitation was measured with sperm motion analysis and an increase in curvilinear velocity (VCL), as the marker of hyperactivated velocity, was observed in all eight cases (p < 0.001, Supplementary Table 1).

**Figure 1:**
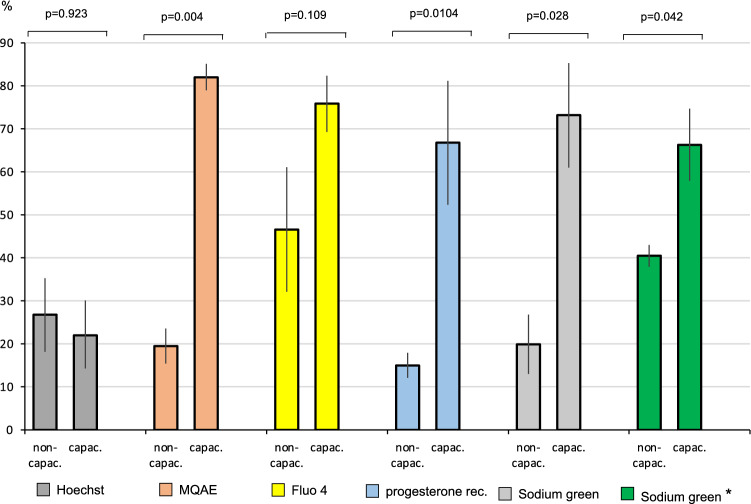
Results FC and sorting of 10,000 or more spermatozoa after incubation in either capacitating or non-capacitating culture medium. The following dyes were used to label the spermatozoa: Hoechst 33342-dye (negative control), MQAE, Fluo4, progesterone receptor-FITC and sodium green (CoroNa^+^). For sorting the spermatozoa were provided by six fertile donors. Three additional sortings were performed using the sodium green dye using spermatozoa provided by three men heterozygous for the Phe508del variant of the cftr-gene (marked with an asterisk).

**Figure 2:**
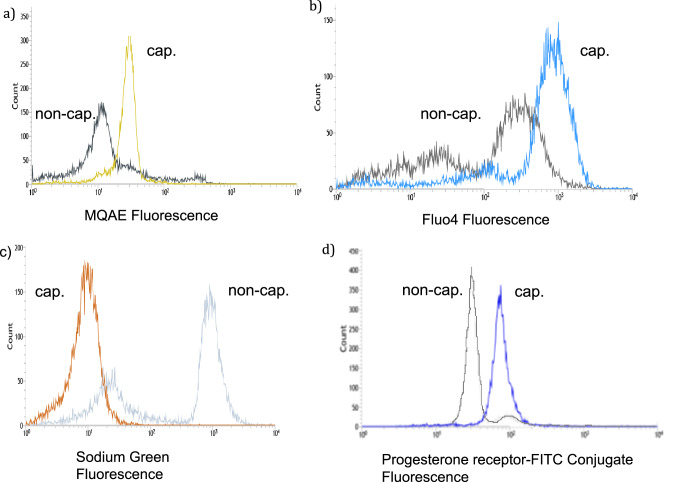
Representative example of FC histograms of capacitating and non-capacitating spermatozoa (donor 3), stained by four different fluorescent dyes. The Y-axis represents the number of spermatozoa labelled by the corresponding fluorescence depicted in the X-axis. (**a**) MQAE: black non-capacitated, yellow capacitated; (**b**) Fluo4: grey non-capacitated, blue capacitated; (**c**) sodium green staining: grey non-capacitated, orange capacitated; (**d**) progesterone-FITC staining: grey non-capacitated, blue capacitated.

Thereafter, the spermatozoa of a total of six normal donors and three primarily infertile men heterozygous for the pathogenic variant of Phe508del were incubated in the same either capacitating or non-capacitating culture medium, stained with five fluorescent dyes and sorted with FACS (Fig. 1). Four of the five dyes have been shown in earlier publications to correlate with capacitation of sperm (see “Methods”). The Hoechst 33342-dye, which has no impact on capacitation, was used as a negative control. Each of the four dyes, MQAE, fluo4, sodium green or progesterone receptor-FITC conjugate, but not the Hoechst 33342-dye, allowed to some degree the distinction between non-capacitated and capacitating spermatozoa (Fig. 2). Using FACS more capacitating than non-capacitating spermatozoa were sorted when labelled by MQAE (p = 0.004), sodium green (p = 0.0104) and based on the progesterone receptor (p = 0.028). Although the number of capacitating spermatozoa could be increased by sorting spermatozoa stained with the fluo4-dye, this difference was statistically not significant because of high variability of the sorting results (p = 0.109). Considering all differences being significant and consistent enough to allow the separation of capacitating spermatozoa from non-capacitating spermatozoa with FACS, the most pronounced difference was achieved with the sodium green dye.

Thus, the sodium green dye was used to distinguish between capacitating and non-capacitating spermatozoa of three men heterozygous for Phe508del (Fig. 1, dark green columns). More spermatozoa were stained with the sodium green dye, when they were incubated in capacitating medium than in non-capacitating medium (p = 0.042). In one patient, the sorted spermatozoa were subjected to single cell PCR to detect the presence of Phe508del. Among the capacitating spermatozoa (weak sodium green signal), 6 out of 38 (16%) spermatozoa carried the Phe508del-variant, 7 out of 38 (18%) did not and in 25 (66%) spermatozoa no signal was detected. Among the non-capacitating spermatozoa (strong sodium green signal), 14/48 (29%) spermatozoa carried the genetic variant, 7/48 (15%) did not and in 27 (56%) spermatozoa no signal was detected.

### Inhibition of capacitation of human spermatozoa through inhibition of CFTR-ion channel

The CFTR-ion channel is known to be involved in the capacitation of sperm^18^. The inhibition of the CFTR-ion channel with the specific inhibitor, CFTTinh-172 (at two different concentrations, 24 µM and 60 µM, respectively) was evaluated both with hyperactivated motion (as given by VCL, µm/s) and through sorting with FACS using five different dyes.

The inhibition of the CFTR-ion channel with CFTRinh-172 on VCL of incubated human spermatozoa, as provided by six normal semen donors, is shown in Supplementary Table 2. The mean curvilinear velocity (VCL), indicating hyperactivation of spermatozoa incubated in capacitating culture medium, decreased significantly when CFTRinh-172 was added to the culture medium (either 24 or 60 μM), indicating CFTR-channel inhibition of hyperactivated velocity as a marker of sperm capacitation in normal semen donors.

**Figure 3:**
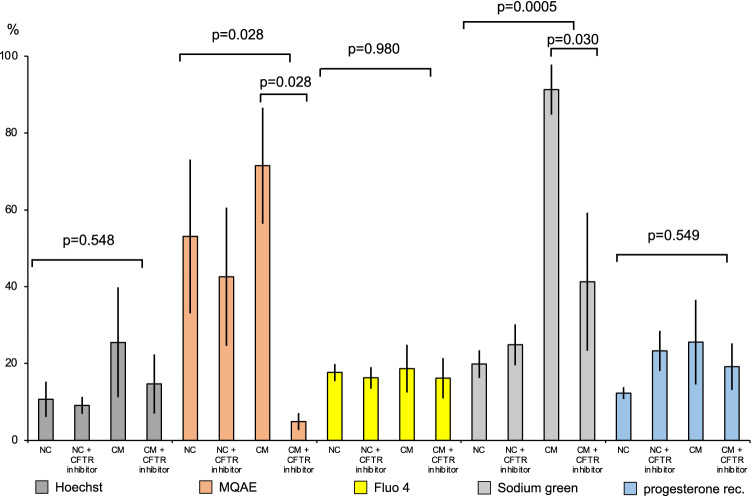
Results of FC sorting of 10,000 or more spermatozoa of five fertile donors after incubation in either capacitating (CM) or non-capacitating (NC) culture medium and in the presence or absence of the CFTR-ion channel inhibitor CFTRinh-172 (24 µM).

A significant inhibition of the calcium ion flow was observed among spermatozoa incubated in capacitating culture medium in the presence of the CFTR-ion channel inhibitor CFTRinh-172 (24 or 60 μM), as given by the fluorescent dye MQAE (p = 0.028) and of sodium ions, as given by the sodium green dye (p = 0.03) (Fig. 3). Moreover, sodium green allowed the detection of a larger number of capacitating spermatozoa in capacitating culture medium, as compared to the number of non-capacitating spermatozoa in non-capacitating culture medium, either with or without the CFTR-ion channel inhibitor (p = 0.0005). However, neither the stimulating effects of the capacitating medium nor the inhibiting effect of the CFTR-inhibitor were detected by fluo4, by staining the progesterone receptor or by the Hoechst 33342-dye, respectively.

### Detection of the CFTR-protein in the membrane of spermatozoa of heterozygous carriers of the Phe508del-variant

**Figure 4:**
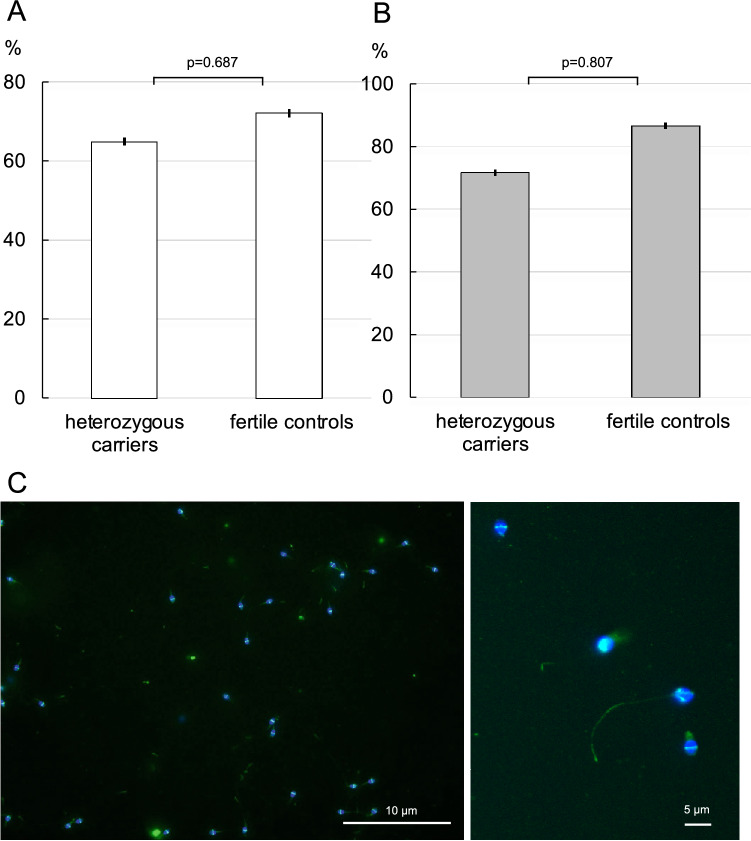
Immunofluorescent staining of the CFTR membrane channel in three heterozygous carriers of the Phe508del-pathogenic variant of the CFTR-gene and three healthy controls demonstrated by similar percentages of spermatozoa with the CFTR protein in the membrane, both in seminal sperm (**A**) and in spermatozoa after swim-up or density gradient centrifugation (**B**). Representative images of spermatozoa stained with the fluorescent anti-CFTR antibody and with the Hoechst 33342-dye (**C**).

Immunofluorescent staining of the CFTR-protein allowed the visualization of the CFTR-ion channel in 64.8% (± 1.7%) of three heterozygous carriers of Phe508del and in 72.1% (± 1.7%) of the spermatozoa of normal healthy controls (Fig. 4A). In samples, prepared with swim-up or density gradient centrifugation with higher motility rates the number of spermatozoa with immunofluorescent staining of the CFTR-protein rose to 76.6% (± 1.7%) in the three Phe508del heterozygous men and to 86.6% (± 1.7%) in the healthy controls (Fig. 4B). The differences in the number of stained spermatozoa between the heterozygous carriers and the controls were statistically not significant. The staining was concentrated in the equatorial segment of the viable spermatozoa, whereas staining was more diffuse or even absent in dead spermatozoa (Fig. 4C). After swim-up or after density gradient centrifugation the number of CFTR-stained spermatozoa was higher than in spermatozoa in semen.

### Genetic transmission of the Phe508del-pathogenic variant to siblings of known heterozygous carriers

**Figure 5:**
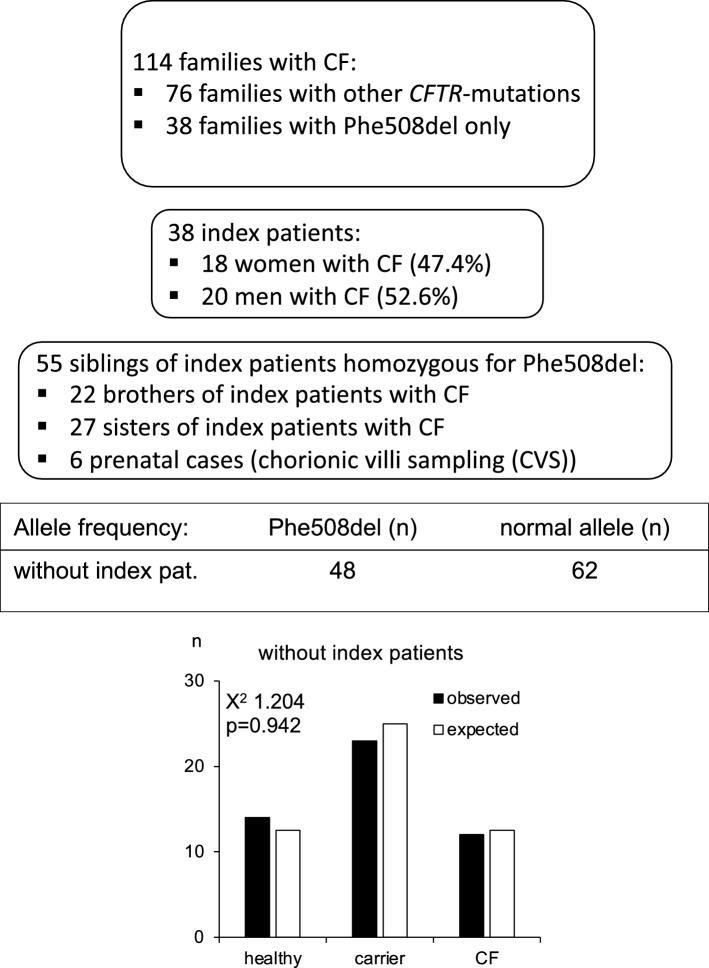
Trio data of 114 families with CF were analysed to demonstrate the inheritance of the genetic trait. In 38 families both parents were carriers of the Phe508del-pathogenic variant leading to at least one child with CF (index patient). Transmission of the mutation to the index patients and to their siblings was analysed (observed) and compared with the Mendelian transmission rate (expected). The distribution of the genotypes was found to correspond to the Hardy–Weinberg equilibrium (X^2^ statistic: 1.204, p = 0.942).

Trio data of 114 families with CF were analyzed to demonstrate the inheritance of the genetic trait (Supplementary Table 3). In 38 families both parents were heterozygous carriers of the Phe508del variant leading to at least one offspring with CF (Fig. 5). All families with pathogenic variants of CFTR other than Phe508del were excluded. Among the 38 index patients with CF 18 were female, 20 were male (Fig. 5). Analysis of trio data resulted in 93 offspring from couples, heterozygous for the Phe508del-pathogenic variant of the *CFTR-*gene (2.45 children per family, ranging from 1 to 6 children). Among the 55 siblings of index patients with CF, 22 were brothers, 27 sisters and 6 cases of unknown gender were prenatally diagnosed by chorionic villi sampling. When the distribution of the genotypes was checked among the siblings of the index patients, their frequencies were found to correspond to the Hardy–Weinberg equilibrium (Chi-squared statistics 1.204, p = 0.942).

## Discussion

Approximately one in 25 individuals is carrier of one pathogenic variants of the *CFTR*-gene, making them the second most frequent recessive disease-causing genetic condition among European-derived individuals. The homozygous condition used to be a lethal disease at an early age, but better medical care has improved survival such that most affected individuals now survive until the age of reproduction and beyond^1,6,23^. Pharmaceutical compounds are now allowing more affected individuals to embark on procreation^13^. Most males with CF are infertile due to CBAVD, whereas the heterozygous condition has been associated with abnormal sperm quality, but these studies have alle been carried out in fertility care institutions^14–16^. Genetic screening for one or more genetic variants of the *CFTR*-gene is increasingly becoming an integral part of infertility care in many institutions offering assisted reproductive technology (ART) services^24^.

The impact of heterozygous variants in the *CFTR-*gene on male fertility remains unclear. The most common pathogenic variant of the *CFTR*-gene, Phe508del, has been in the European gene pool for more than 30,000 years^4,5^ and it is not conceivable why such a frequent and potentially lethal genetic condition is so prevalent in a population if it in addition to mortality contributes to infertility. Earlier studies demonstrating a higher prevalence of genetic variants among infertile men may have been biased while most if not all these studies were carried out in specialized fertility care providing institutions^25^. During sperm transit in the female genital tract the defective CFTR-ion channels of the spermatozoa of heterozygous carriers are compensated by the normal CFTR-function in the female tissues^26^.

Through the measurement of the inward flow of chloride and bicarbonate ions and outward flow of sodium ions in capacitating spermatozoa and through the selective inhibition of the CFTR-ion channel, we here demonstrate the involvement of CFTR in the capacitation of human spermatozoa. Immunofluorescence demonstrated that most spermatozoa of heterozygous carriers of the Phe508del-variant and of healthy controls displayed the CFTR-protein in their membrane and that the numbers of spermatozoa were higher after swim-up or density gradient centrifugation. The functionality of the CFTR-protein in the spermatozoa of the heterozygous carriers was demonstrated by similar rates of capacitation, as given by FC and by similar numbers of spermatozoa with or without the genetic condition in all fractions sorted with FACS. We conclude that the capacitation of mature sperm of heterozygous men carrying the Phe508del-variant is not affected by the disorder.

*CFTR* is expressed in Sertoli cells^27,28^ and has been shown to be involved in spermatogenesis^29^. All cells in the seminiferous tubules share their transmembrane proteins during spermatogenesis^30^. In the mouse model it was demonstrated that during spermatogenesis the sharing of gene products result in genetically distinct spermatids becoming phenotypically equivalent^31,32^. Intimate contacts between Sertoli cells and germ cells, as mediated by junctional membranes and lipid droplet contact, may well be involved in sharing healthy CFTR-ion channels during spermatogenesis.

Based on these findings and to further evaluate the fertility of heterozygous carriers of Phe508del, we analyzed the trio data of 38 families with CF specifically carrying the Phe508del-variant. The heterozygous parents of index patients with CF had between one and six children (mean number of children: 2.45). After exclusion of the index patients the distribution of the alleles among the siblings of the index patients with CF did not differ from the expected Mendelian inheritance pattern, suggesting normal fertility of heterozygous carriers of the pathogenic variant. These findings are in agreement with earlier findings indicating normal or even better fertility among heterozygous carriers of the Phe508del-variant in the Netherlands and in Denmark^33,34^.

Until recent years CF was lethal at an early age, well before the age of reproduction. Using the Hardy–Weinberg equation early lethality in the homozygous state would result in a rapid disappearance of the pathogenic variant within few generations. Instead, the Phe508del-pathogenic variant of the *CFTR*-gene has been prevalent among Europeans for at least 30,000 years. If one human generation needs 22 to 33 years for reproduction, Phe508del has persisted for up to 900 to 1300 generations. Deviations from the Hardy–Weinberg equilibrium occur in small populations causing genetic drift. Genetic drift may perhaps be responsible for the higher incidence of Phe508del in previously isolated communities^4^ but does not explain its high prevalence throughout Central and Northern Europe. Deviations from the Hardy–Weinberg equilibrium may also result from balanced selection in favour of the variant, possibly due to protection against historic pandemic infections, such as chronic diarrhea^35,36^, cholera^37,38^, thyphus^39^, tuberculosis^40,41^, including even syphilis^42^ and bubonic plague^43^. Historically, relative protection against those pandemic infections together with unimpaired fertility may have allowed carriers of the Phe508del-variant to produce more offspring^33^.

In conclusion, inhibition of the CFTR membrane channel interferes with the capacitation of spermatozoa. Most viable spermatozoa from three infertile men heterozygous for Phe508del carry the functional ion channel and sperm capacitation appears similar as in normal controls. Family data of Phe508del heterozygous couples do not point towards any impairment of fertility in heterozygous individuals carrying the Phe508del-variant.

## Methods

This study was presented to and approved by the Ethics Committee of Northwestern Switzerland (EKNZ 219/13) and informed consent was obtained from all participating individuals. The authors confirm that all experiments were performed in accordance with relevant named guidelines and regulations.

This experimental study was carried out with semen of a total of nine healthy semen donors (in two sets of experiments) and of three heterozygous men, all three carrying the Phe508del-pathogenic variant and originally presenting with infertility. Phe508del was identified from a panel of more than 50 pathogenic variants of the cftr-gene, representing 92% of the most common variants in Switzerland. Among the three men carrying Phe508del the causes of infertility were either unexplained, or due to tubal occlusion or endometriosis, respectively. In all men the concentration of the spermatozoa in the seminal plasma was normal, so that FACS of their spermatozoa was possible. All eventually fathered children, one after ICSI, one after IVF and one after natural conception.

All participating individuals were informed about the rationale of the study and signed an informed consent form prior to their participation. In addition, a retrospective assembly of the trio data of 114 families with known pathogenic variant(s) of the cftr-gene was also approved by the ethics committee as a later amendment to the original research proposal.

### Conventional semen analysis and semen preparation

Conventional semen analysis was performed following the WHO-guidelines (2010). The andrology laboratory underlies both an internal and an external quality control (QuaDeGa). Routine semen analysis includes sperm count (millions per ml), normal morphology (%) and progressive and total motility (%). After conventional semen analysis, semen was prepared using the swim-up protocol or with density gradient centrifugation^44^. Hyperactivated sperm motion as a marker of sperm capacitation was measured with computerized sperm image analysis (SCA)^45^. The gating was validated and set as follows: sperm head length 4.1–5.1 µm, width 2.6–3.6 µm, surface 9.1–15.6 µm^2^, perimeter 11.0–15.9 µm, ellipticity 1.3–1.8 and elongation 0.12–0.30; sperm midpiece width 0.10–1.50 µm; sperm tail length 12.0–100.0 µm. Images were acquired at 60 Hz.

### Discrimination between capacitated and non-capacitated spermatozoa using FACS

Semen samples of six fertile semen donors with normal semen characteristics as given by conventional semen analysis and three men carrying the Phe508del-variant were analyzed to discriminate between capacitating and non-capacitating spermatozoa with FC and were divided into two aliquots and subsequently prepared with swim-up in Sydney IVF-medium (Cook Medical). In each experiment 10′000 spermatozoa or more were analyzed. For the inhibition of capacitation half of the sample was incubated in culture medium lacking both serum albumin and bicarbonate preventing the onset of capacitation^46^. The other half of the sample was treated with culture medium containing recombinant human albumin (5 mg/ml, Sigma-Aldrich, Buchs, Switzerland), sodium-bicarbonate (25 mM, Sigma-Aldrich) and membrane-permeable dbcAMP (1 mM, Sigma-Aldrich, Buchs, Switzerland) induce capacitation^46^. To confirm capacitation the samples were evaluated by computerized sperm motion analysis to demonstrate hyperactivated motility, as given by an increase in curvilinear velocity (VCL^45^).

In order to demonstrate capacitation the CoroNa^+^ dye (sodium green) was used to visualize the presence of intracellular sodium^47^, the N-Ethoxycarbonylmethyl-6-methoxyquinolinium bromide-dye for the presence of the intracellular chloride (MQAE^48^), the fluo4-dye for the presence of intracellular calcium ions^49^, the merocyanine 540-dye for the composition of membrane lipidic^43^ and progesterone 3-(O-carboxymethyl)oxime:BSA–fluorescein isothiocyanate conjugate for the demonstration of the progesterone receptor^50^. A subset of spermatozoa was also stained with the Hoechst 33342-dye (BD Biosciences), used as a negative control unrelated to capacitation.

### Inhibition of CFTR ion channel

In five healthy semen donors the capacitation of washed spermatozoa was inhibited by blocking the CFTR-ion channel with the CFTR-inhibiting substance dyphenyl-amine-2-carboxylate (DPC, CFTRinh-172, Sigma-Aldrich), used at two different concentrations, 24 µM and 60 µM, respectively^51,52^. In aliquots containing either normal or CFTR-inhibited spermatozoa capacitation was induced by adding human serum albumin and bicarbonate ions and capacitation was visualized both with sperm motion analysis and with FC using each of the five different staining methods listed above.

### Immunofluorescence of CFTR in sperm of three heterozygous carriers of Phe508del

Spermatozoa were stained with a primary anti-CFTR-antibody and a secondary, fluorescently labelled (fluorescein isothiocyanate, FITC) rabbit polyclonal antibody was used (Abcam, ab131553) to demonstrate the presence of the CFTR ion channel in the membrane of spermatozoa of three men, known to be heterozygous carriers of the Phe508del-variant. The CFTR-protein was detected as a brightly green-fluorescent band in the equatorial region of the spermatozoa heads. The Hoechst 33342-dye was used to stain the nucleus of the spermatozoa with a blue color. All native semen samples were analyzed in triplicate and those containing washed spermatozoa in duplicate or triplicate, depending on the number of spermatozoa. At least 150 spermatozoa were evaluated in each slide. The slides were analyzed under a fluorescence microscope Olympus BX63 Apollo using the software CellSens. The images were processed with the FIJI (ImageJ) image-processing program.

### Detection of Phe508del with PCR in single spermatozoa after sorting with FACS

The sodium-green sorted samples of one heterozygous carrier of Phe508del containing either capacitated or non-capacitated spermatozoa were washed and diluted to 1 cell/μl. Single spermatozoa were sorted into single wells of 96-well plates. In half of each plate (48 wells) one spermatozoon per well was sorted out of the blue encircled region (high fluorescence). In the other half plate one single spermatozoon per well was sorted from the violet encircled region (low fluorescence). For positive control of single cell PCR fibroblasts containing the Phe508del-variant were used (Coriell Institute, GM00998).

Lysis was obtained by a freshly prepared lysis solution and terminated by a neutralizing buffer. Whole genomic amplification was achieved by multiple displacement amplification, following the manufacturer’s manual (all by Qiagen Inc., REPLI-g kit). After having pre-amplified the aliquots, dilutions of 1/50 were used for further sequencing. Three different primers for the cftr-gene were used to detect Phe508del. The normal gene primers used were 5′-GGCACCATTAAAGAAAATATCATCTTTG-3′ and 5′-AGCTTCTTAAAGCATAGGTCATGTG-3′. The Phe508del primer used was 5′-CTGGCACCATTAAAGAAAATATCATTG-3′. One served as a positive control to confirm amplification of the cftr-gene and two as controls to identify or exclude the presence of Phe508del, respectively.

### Statistical analyses

All FACS experiments were carried out at least in triplicates. Statistical analyses of potential differences were carried out with ANOVA and Tukey’s test or with the X^2^-test of observed and expected values^53^. The level of statistical significance was set at the 5% level.

## Supplementary Information

Supplementary Table 1.

Supplementary Table 2.

Supplementary Table 3.
