# Supplementary material for: Identification and selection of healthy spermatozoa in heterozygous carriers of the Phe508del-variant of the CFTR-gene in assisted reproduction
Source: Sci Rep. 2022 Feb 3;12:1866. doi: 10.1038/s41598-022-05925-1 (PMC8814069; doi:10.1038/s41598-022-05925-1)
Supplement: Supplementary file 1 — Supplementary Table 1. [file 41598_2022_5925_MOESM1_ESM.pdf]

# Identification and selection of healthy spermatozoa in heterozygous carriers of the Phe508del-variant of the CFTR-gene in assisted reproduction

Julie De Geyter[1], Sabina Gallati-Kraemer[3], Hong Zhang[4], Christian De Geyter

**Supplementary Table 1**

The effect of incubating prepared spermatozoa in non-capacitating culture medium and in capacitating culture medium on hyperactivated sperm motion, as given by curvilinear velocity (VCL). The semen samples were donated by eight normal donors.

| Donor | VCL (µm/sec.), non-capacitating | VCL (µm/sec.), capacitating |
|---|---|---|
| 1 | 50.0 | 125.0 |
| 2 | 38.0 | 61.2 |
| 3 | 37.2 | 93.6 |
| 4 | 25.3 | 100.7 |
| 5 | 47.0 | 78.0 |
| 6 | 49.7 | 94.7 |
| 7 | 37.4 | 105.0 |
| 8 | 38.7 | 97.5 |
| mean | 40.4 | 94.5 |
| SD | 8.3 | 18.8 |

p=0.000105 (ANOVA)
