# Supplementary material for: Identification and selection of healthy spermatozoa in heterozygous carriers of the Phe508del-variant of the CFTR-gene in assisted reproduction
Source: Sci Rep. 2022 Feb 3;12:1866. doi: 10.1038/s41598-022-05925-1 (PMC8814069; doi:10.1038/s41598-022-05925-1)
Supplement: Supplementary file 2 — Supplementary Table 2. [file 41598_2022_5925_MOESM2_ESM.pdf]

Identification and selection of healthy spermatozoa in heterozygous carriers of the Phe508del-variant of the CFTR-gene in assisted reproduction

Julie De Geyter[1], Sabina Gallati-Kraemer[3], Hong Zhang[4], Christian De Geyter

Supplementary Table 2

The inhibition of the CFTR-ion channel immediately after wash, during incubation in capacitating culture medium, in capacitating culture medium supplemented with CFTRinh-172 (either 24 µM or 60 µM) on VCL (in µm/sec.) of incubated human spermatozoa of six normal semen donors.

Donor	after first wash	capacitating med.	+CFTR inh (24 µM)	+CFTR inh (60 µM)
1	47.8	78.1	30.9	34.1
2	27.0	61.5	26.9	34.6
3	49.7	94.7	65.0	49.8
4	35.0	116.0	57.1	58.7
5	37.4	105.0	42.5	42.5
6	38.7	97.5	66.4	27.7
Mean	39.3	92.1	48.1	41.3
SD	8.4	19.5	17.2	11.4
p=0.00001 (ANOVA)				
