# Supplementary material for: Identification and selection of healthy spermatozoa in heterozygous carriers of the Phe508del-variant of the CFTR-gene in assisted reproduction
Source: Sci Rep. 2022 Feb 3;12:1866. doi: 10.1038/s41598-022-05925-1 (PMC8814069; doi:10.1038/s41598-022-05925-1)
Supplement: Supplementary file 3 — Supplementary Table 3. [file 41598_2022_5925_MOESM3_ESM.pdf]

Identification and selection of healthy spermatozoa in heterozygous carriers of the Phe508del-variant of the CFTR-gene in assisted reproduction

Julie De Geyter, Sabina Gallati-Kraemer, Hong Zhang, Christian De Geyter

Supplementary Table 3:

Trio data of 114 families with CF were analysed to demonstrate the inheritance of the genetic trait, among which 38 families with both parents being carriers of the Phe508del-pathogenic variant leading to at least one offspring with CF. In pink are marked the index patients, in blue homozygous Phe508del patients.

Fam No	Patient No	Family members	Gender	Paternal Mutation	Maternal Mutation
1	1.1		female	Phe508del	Phe508del
1	1.2	Brother		Phe508del	wt
1	1.3	or		wt	Phe508del
2	2.1		male	N1303K	2789+5G>A
2	2.2	Brother		wt	2789+5G>A
2	2.3	Sister		wt	2789+5G>A
2	2.4	Uncle p	male	wt	
2	2.5	Aunt p		N1303K	
2	2.6	Aunt m			wt
3	3.1		female	Phe508del	Phe508del
3	3.2	Sister		wt	Phe508del
3	3.3	Uncle p		wt	
4	4.1		female	Phe508del	Phe508del

4	4.2	Sister			Phe508del	wt
4		or			wt	Phe508del
5	5.1			male	Phe508del	Phe508del
5	5.2	Sister			Phe508del	Phe508del
5	5.3	Brother			wt	wt
5	5.4	Aunt p			Phe508del	
6	6.1			male	Phe508del	Phe508del
6	6.2	Brother			Phe508del	wt
6			or		wt	Phe508del
6	6.3	Brother			Phe508del	wt
6			or		wt	Phe508del
6	6.4	Uncle m				wt
7	7.1			male	Phe508del	Phe508del
7	7.2	Brother			Phe508del	wt
7			or		wt	Phe508del
8	8.1			male	Phe508del	3905insT
8	8.2	Brother			Phe508del	wt
8	8.3	Sister			wt	wt
8	8.4	Aunt m				3905insT
8	8.5	Uncle m				wt
8	8.6	Aunt p			Phe508del	
9	9.1			male	621+1G>T	Phe508del
9	9.2	Brother			wt	wt
10	10.1			male	Phe508del	Phe508del
10	10.2	Brother			wt	wt
11	11.1			female	L1065P	N1303K
11	11.2	Sister			wt	wt
12	12.1			female	3905insT	1898+1G>A

12	12.2	Sister		3905insT	wt
12	12.3	Aunt m			1898+1G>A
13	13.1		female	Phe508del	Phe508del
13	13.2	Sister		wt	wt
14	14.1		female	3905insT	P5L
14	14.2	Sister		wt	P5L
15	15.1		female	Phe508del	Deletion
16	16.1		female	Phe508del	Phe508del
16	16.2	Sister (twin)		Phe508del	Phe508del
16	16.3	Uncle p		wt	
17	17.1		male	Phe508del	Deletion
17	17.2	Sister		wt	wt
17	17.3	Brother (twin)		Phe508del	Deletion
17	17.4	Brother (twin)		Phe508del	Deletion
18	18.1		female	Phe508del	Phe508del
18	18.2	Sister		Phe508del	wt
18		or		wt	Phe508del
18	18.3	Brother		wt	wt
19	19.1		female	Phe508del	Phe508del
19	19.2	Sister		wt	Phe508del
19	19.3	Sister		wt	wt
20	20.1		male	Phe508del	Phe508del
20	20.2	Sister		wt	wt
20	20.3	Sister		Phe508del	wt
20	20.4	Brother		Phe508del	Phe508del
20	20.5	Uncle m			Phe508del
20	20.6	Aunt p		Phe508del	
20	20.7	Cousin p		Phe508del	Phe508del

20	20.8	Cousin p			Phe508del	wt
20			or		wt	Phe508del
21	21.1			female	Phe508del	G542X
21	21.2	Sister			Phe508del	wt
22	22.1			male	G85E	Phe508del
22	22.2	Sister			G85E	wt
23	23.1			female	G85E	Phe508del
24	24.1			male	Phe508del	1717-1G>A
24	24.2	Sister			wt	1717-1G>A
25	25.1			male	3849+10kbC>T	1717-1G>A
25	25.2	Sister			3849+10kbC>T	1717-1G>A
25	25.3	Sister			3849+10kbC>T	wt
25	25.4	Sister			3849+10kbC>T	1717-1G>A
25	25.5	Brother			wt	wt
26	26.1			male	Phe508del	Phe508del
26	26.2	Sister			Phe508del	wt
26			or		wt	Phe508del
27	27.1			female	Phe508del	N1303K
27	27.2	Cousin m			3905insT	N1303K
28	28.1			male	Phe508del	3905insT
28	28.2	Brother			Phe508del	wt
29	29.1			female	Phe508del	Phe508del
29	29.2	Brother			wt	wt
30	30.1			male	Phe508del	Phe508del
30	30.2	Brother			wt	Phe508del
31	31.1			female	Phe508del	2176insC
31	31.2	Brother			wt	2176insC
31	31.3	Sister			Phe508del	wt

31	31.4	Uncle m		wt	wt
31	31.5	Aunt m		wt	wt
32	32.1		male	W1282X	Phe508del
32	32.2	Brother		W1282X	wt
33	33.1		female	R1162X	Phe508del
33	33.2	Brother		R1162X	wt
33	33.3	Niece		wt	wt
34	34.1		male	Phe508del	Phe508del
34	34.2	Sister		wt	wt
35	35.1		female	Q525X	Phe508del
35	35.2	Uncle m		wt	Phe508del
36	36.1		female	Phe508del	Phe508del
36	36.2	Sister		Phe508del	wt
36		or		wt	Phe508del
37	37.1		female	Phe508del	Phe508del
37	37.2	Sister		Phe508del	wt
37		or		wt	Phe508del
37	37.3	Brother		Phe508del	wt
37		or		wt	Phe508del
37	37.4	Nephew		wt	Phe508del
37	37.5	Nephew		wt	Phe508del
37	37.6	Niece		Phe508del	Phe508del
39	39.1		male	3905insT	3905insT
39	39.2	Sister		3905insT	wt
39		or		wt	3905insT
40	40.1		female	Phe508del	Phe508del
40	40.2	Sister		wt	Phe508del
41	41.1		male	Phe508del	Phe508del

41	41.2	Sister		wt	wt
42	42.1		male	Phe508del	Phe508del
42	42.2	Sister		Phe508del	wt
42		or		wt	Phe508del
43	43.1		male	R553X	R553X
43	43.2	Aunt p		R553X	
43	43.3	Cousin p		R553X	
44	44.1		male	Phe508del	Phe508del
44	44.2	Sister		Phe508del	Phe508del
44	44.3	Brother		Phe508del	Phe508del
44	44.4	Sister		Phe508del	Phe508del
44	44.5	Brother		Phe508del	wt
44		or		wt	Phe508del
44	44.6	Sister		wt	Phe508del
45	45.1		female	Phe508del	Phe508del
45	45.2	CVS		wt	wt
45	45.3	CVS		wt	wt
45	45.4	CVS	female	Phe508del	wt
45		or		wt	Phe508del
46	46.1		male	Phe508del	W1282X
47	47.1		female	Phe508del	Phe508del
47	47.2	Brother (twin)		wt	wt
48	48.1		female	Phe508del	1717-1G>A
48	48.2	CVS		wt	wt
49	49.1		female	1717-1G>A	1717-1G>A
49	49.2	Sister		wt	wt
50	50.1		female	I507del	Phe508del
51	51.1		female	G542X	TG12_T5

51	51.2	Sister			G542X	TG12_T5
51	51.3	Brother			G542X	TG12_T5
51	51.4	Brother			wt	wt
52	52.1			male	Phe508del	Phe508del
52	52.2	Brother			Phe508del	wt
52			or		wt	Phe508del
52	52.3	Sister			wt	wt
53	53.1			female	M1101K	M1101K
53	53.2	Sister			M1101K	M1101K
53	53.3	Brother			M1101K	M1101K
54	54.1			male	Phe508del	Phe508del
54	54.2	Sister			Phe508del	Phe508del
55	55.1			female	Phe508del	Phe508del
55	55.2	Brother			Phe508del	wt
55			or		wt	Phe508del
56	56.1			female	Phe508del	Phe508del
56	56.2	Sister			Phe508del	wt
56			or		wt	Phe508del
57	57.1			male	Phe508del	Phe508del
57	57.2	Uncle m				Phe508del
57	57.3	Cousin m				Phe508del
58	58.1			female	Phe508del	Phe508del
58	58.2	CVS			wt	wt
58	58.3	CVS			Phe508del	wt
58			or		wt	Phe508del
58	58.4	Cousin p			Phe508del	
59	59.1			male	Del. 14b-17b	P5L
59	59.2	Sister			Del. 14b-17b	P5L

60	60.1		female	G542X	Phe508del
60	60.2	Brother		wt	Phe508del
61	61.1		female	1525-1G>A	1525-1G>A
61	61.2	CVS		wt	wt
62	62.1		male	1717-1G>A	711+5G>A
63	63.1		male	Phe508del	Phe508del
63	63.2	Brother		Phe508del	Phe508del
64	64.1		male	Phe508del	Phe508del
64	64.2	Brother		wt	Phe508del
65	65.1		female	420del9	Phe508del
65	65.2	Sister		420del9	wt
65	65.3	Nephew		420del9	
66	66.1		male	R553X	W1089X
66	66.2	AZ		R553X	W1089X
67	67.1		female	Del. 17a-17b	Phe508del
68	68.1		male	Phe508del	Phe508del
68	68.2	Brother (twin)		Phe508del	Phe508del
68	68.3	Brother		Phe508del	Phe508del
68	68.4	Sister		Phe508del	Phe508del
68	68.5	Brother		wt	wt
68	68.6	Brother		wt	wt
69	69.1		male	Phe508del	G178R
69	69.2	CVS		Phe508del	G178R
69	69.3	CVS		Phe508del	wt
69	69.4	CVS		Phe508del	G178R
70	70.1		male	TG13_T5	Phe508del
70	70.2	Sister		wt	Phe508del
71	71.1		female	Q39X	N1303K

72	72.1		female	G542X	H1054D
72	72.2	Brother		wt	H1054D
73	73.1		female	Phe508del	Q525X
73	73.2	Brother		Phe508del	wt
74	74.1		female	Phe508del	Phe508del
74	74.2	Sister		Phe508del	wt
74		or		wt	Phe508del
75	75.1		male	R347H	Phe508del
75	75.2	Brother		wt	wt
76	76.1		female	3905insT	Phe508del
77	77.1		male	TG12_T5	3905insT
77	77.2	Sister		TG12_T5	3905insT
78	78.1		male	Phe508del	Phe508del
78	78.2	Sister		wt	wt
79	79.1		female	Phe508del	K68X
79	79.2	Sister		Phe508del	K68X
80	80.1		male	1717-1G>A	1717-1G>A
80	80.2	Sister		wt	wt
80	80.3	Sister		wt	wt
81	81.1		female	Phe508del	3905insT
81	81.2	Aunt p		Phe508del	
81	81.3	Uncle m			3905insT
81	81.4	Cousin p		Phe508del	
82	82.1		male	Phe508del	2789+5G>A
83	83.1		female	E585X	Phe508del
83	83.2	CVS		wt	Phe508del
84	84.1		female	2347delG	Phe508del
85	85.1		female	Phe508del	2347delG

85	85.2	CVS			wt	wt
86	86.1			male	Phe508del	W1282X
87	87.1			male	Phe508del	Phe508del
87	87.2	Sister			wt	wt
87	87.3	Aunt m				wt
88	88.1			female	1717-1G>A	Phe508del
88	88.2	Aunt m				Phe508del
89	89.1			female	3272-26A>G	Phe508del
90	90.1			female	W1282X	R347H
91	91.1			female	Phe508del	Phe508del
91	91.2	CVS		female	Phe508del	wt
91			or		wt	Phe508del
92	92.1			male	Phe508del	Phe508del
93	93.1			female	Phe508del	R347P
94	94.1			male	Phe508del	R75X
94	94.2	CVS			Phe508del	wt
95	95.1			male	1717-1G>A	Phe508del
95	95.2	Uncle p			wt	
96	96.1			female	Phe508del	1717-1G>A
97	97.1			male	Phe508del	2143delT
97	97.2	Sister			Phe508del	2143delT
98	98.1			male	Phe508del	R553X
99	99.1			male	2183AA>G	2184insA
100	100.1			male	Phe508del	D1152H
100	100.2	Brother			wt	D1152H
101	101.1			male	Phe508del	3905insT
102	102.1			male	Phe508del	E528K
103	103.1			female	TG12_T5	Phe508del

103	103.2	Brother		TG12_T5	wt
104	104.1		male	Phe508del	N1303K
105	105.1		female	3180delA	N1303K
106	106.1		female	Phe508del	R553X
107	107.1		female	R1162X	R347H
108	108.1		female	3905insT	R1066H
109	109.1		female	935delA	3272-26A>G
110	110.1		female	Q525X	R347P
110	110.2	Brother		wt	wt
111	111.1		female	R553X	Phe508del
111	111.2	Sister		R553X	Phe508del
112	112.1		male	3229_3230delCT	3229_3230delCT
112	112.2	CVS		wt	wt
113	113.1		female	R334W	Phe508del
114	114.1		male	Phe508del	R347H
